# Circular RNA hsa_circ_0061825 (circ‐TFF1) contributes to breast cancer progression through targeting miR‐326/TFF1 signalling

**DOI:** 10.1111/cpr.12720

**Published:** 2020-01-21

**Authors:** Gaofeng Pan, Anwei Mao, Jiazhe Liu, Jingfeng Lu, Junbin Ding, Weiyan Liu

**Affiliations:** ^1^ Minhang Hospital Fudan University Shanghai China

**Keywords:** breast cancer, circ‐TFF1, miR‐326, TFF1

## Abstract

**Objectives:**

Circular RNAs (circRNAs) are RNA transcripts that belong to non‐coding RNAs (ncRNAs), whose implication in human cancers has been recently demonstrated. However, the specific role of multiple circRNAs in breast cancer remains unidentified.

**Materials and methods:**

Microarray analysis and bioinformatics analysis were applied to select circRNA and miRNA, respectively. The loop structure of circ‐TFF1 was confirmed using RNase R treatment, divergent primer PCR and Sanger sequencing. qRT‐PCR and Western blot were employed for gene expressions. In vitro and in vivo experiments were conducted to assess the function of circ‐TFF1 in biological processes in breast cancer cells. FISH and subcellular separation indicated circ‐TFF1 cellular distribution. Luciferase reporter and RIP assays and Pearson's correlation analysis were performed to evaluate relationships between genes.

**Results:**

Circ‐TFF1 and TFF1 were both upregulated and positively associated with each other in breast cancer. Knockdown of circ‐TFF1 hindered breast cancer cell proliferation, migration, invasion and EMT in vitro and controlled tumour growth in vivo. Circ‐TFF1 acted as a ceRNA of TFF1 by sponging miR‐326, and its contribution to breast cancer progression was mediated by miR‐326/TFF1 axis.

**Conclusions:**

Circ‐TFF1 is a facilitator in breast cancer relying on TFF1 by absorbing miR‐326, providing a novel promising target for BC treatment.

## 1 INTRODUCTION

Breast cancer is one of the most frequent malignancies and the leading cause of cancer‐related mortality in women all around the world.^1^ Despite numerous diagnostic and therapeutic improvements, the recurrence rate and survival rates of women with breast cancer are still disappointing.^2^, ^3^ Therefore, to provide more effective diagnostic or therapeutic strategies, the sustained efforts are of great necessity to understand key molecules implicated in the onset and development of breast cancer.

Circular RNAs (circRNAs) are new members of non‐coding RNAs (ncRNAs) that form a loop with jointed 3′ heads and 5′ tails.^4^ Due to their wide expression in mammals,^5^ the role and potential functions of circRNAs in human diseases attached the attention of most scientists.^6^ Currently, several reports also described the role of a few circRNAs in breast cancer.^7^ For example, circEPSTI1 is a prognostic marker and regulator in triple‐negative breast cancer (TNBC).^8^ CircTADA2As inhibit breast cancer progression and metastasis through miR‐203a‐3p/SOCS3 axis.^9^ CircRNA hsa_circ_001783 affects breast cancer progression by sponging miR‐200c‐3p.^10^ Hsa_circRNA_0006528 functions as a competing endogenous RNA (ceRNA) to facilitate breast cancer progression via miR‐7‐5p/MAPK/ERK pathway.^11^ CircANKS1B promotes breast cancer cell metastasis by releasing miR‐148a/152‐3p‐silenced USF1.^12^ However, more effective circRNAs that participate in breast cancer pathogenesis and progression need to be recognized to maximize therapeutic effectiveness.

In the present study, we uncovered a novel circRNA called hsa_circ_0061825 (circ‐TFF1) as a contributor in breast cancer progression. Circ‐TFF1 is derived from the host gene trefoil factor 1 (TFF1) that locates in 21q22.3, and its tumour‐facilitating role in breast cancer was illustrated to be mediated by TFF1, an oncogene identified by accumulating reports.^13^, ^14^, ^15^, ^16^ Intriguingly, circ‐TFF1 positively modulated TFF1 expression in breast cancer through competitively binding with miR‐326, a tumour‐suppressive gene in various cancers 17, 18, 19 and a growth‐inhibitory miRNA in breast cancer.^20^, ^21^ Our findings in this study unveiled a tumour‐promoting network of circ‐TFF1/miR‐326/TFF1 axis in breast cancer, providing a promising biomarker for future treatment of patients with breast cancer.

## 2 MATERIALS AND METHODS

### 2.1 Sample collection of breast cancer tissues

The breast cancer tissues, adjacent normal tissues and healthy breast tissues required this paper obtained from Minhang Hospital, Fudan University and immediately after being cut‐off went through frozen preservation, in the tubes supplemented with the RNA‐later preservative, and well maintained in liquid nitrogen. Patients where all the tissues derived from were informed and permitted with a written consent, and none of these enrolled patients received any treatments prior to this. With approval from the Ethics committee of Minhang Hospital, Fudan University, the current study was conducted.

### 2.2 Cell lines and cell culture

Required breast cancer cell lines (MCF‐7, MDA‐MB‐453, BT‐549, MDA‐MB‐231) and corresponding normal breast cells (MCF‐10A) acquired from the American Type Culture Collection (ATCC; Manassas, VA, USA) was recommended to cultivate in Dulbecco's Modified Eagle's Medium (DMEM; Invitrogen, Carlsbad, CA, USA) in moist air with 5% CO_2_ at 37°C. 10% foetal bovine serum (10% FBS; Cat. No: 10099‐141; Gibco; Grand Island, NY, USA) and 100 U/mL penicillin/streptomycin (Cat. no: 15140148; Invitrogen, Shanghai, China) were also needed in the media.

### 2.3 Cell transfection

To silence circ‐TFF1, miR‐326 and TFF1, sh‐circ‐TFF1#1/2/3, miR‐326 inhibitor and sh‐TFF1#1 specifically targeting circ‐TFF1, miR‐326 and TFF1 together with their respective controls (sh‐NC, miRNA NC, sh‐NC) were synthesized and commercially purchased from GenePharma (Shanghai, China). For the downregulation of miR‐326, miR‐326 inhibitor (GenePharma; Shanghai, China) targeting miR‐326 and its negative control were also applied. Transfection of these well‐established plasmids into breast cancer cells was implemented following the instruction of Lipofectamine 2000 reagent (Cat. no: 11668019; Invitrogen; Carlsbad, CA, USA) at a ratio of 1:4.

### 2.4 Real‐time quantitative PCR (qRT‐PCR)

To assess the expression profile of circ‐TFF1, TFF1, miR‐326, miR‐889‐3p, miR‐4731‐5p, miR‐2278, miR‐330‐5p in the breast cancer tissues or its cells, qRT‐PCR was carried out with several kits employed. Firstly, Trizol (Cat. no: 15596‐026; Invitrogen, Carlsbad, CA, USA) extracted total RNAs from tissues or cells with PrimeScript RT Reagent Kit (Cat. no: HRR037A; TaKaRa; Dalian, Liaoning, China) aimed for cDNA synthesis. Secondly, ABI StepOne™ Real‐Time PCR Systems (Cat. no: TRN00076; Applied Biosystems; Carlsbad, CA, USA) analysed their expression levels in breast cancer tissues or cells using 2^‐ΔΔCT^ method. Lastly, the reference gene for lncRNA and mRNA is GAPDH, while five miRNAs were normalized to U6.

### 2.5 Western blot

After RIPA protein extraction reagent (P0013; Beyotime; Beijing, China) supplied with phenylmethylsulfonyl fluoride and protease inhibitor treatment, extracted proteins (20 μg) from transfected cells were determined as for its protein density and went through separation with 10% SDS‐PAGE, followed by immediate transfer onto a PVDF membrane (Cat. no: 3010040001; Roche; Shanghai, China). Separated proteins went through an overnight incubation with anti‐Bcl‐2 (1:1000 dilution; ab182858; abcam), anti‐Bax (1:1000 dilution; ab182733; abcam), anti‐cleaved caspase‐3 (1:1000 dilution; ab2302; abcam), anti‐caspase‐3 (1:1000 dilution; ab13585; abcam), anti‐E‐cadherin (1:1000 dilution; ab1416; abcam), anti‐N‐cadherin (1:1000 dilution; ab18203; abcam), anti‐MMP2 (1:1000 dilution; ab37150; abcam), anti‐MMP9 (1:1000 dilution; ab38898; abcam), anti‐TFF1 (1:1000 dilution; ab92377; abcam), anti‐GADPH (1:1000 dilution; ab8245; abcam) at 4°C and 1 hour incubation with Rabbit Anti‐Mouse IgG H&L (HRP) (1:2000 dilution; ab6728; abcam) in 5% non‐fat milk at indicated dilution. Densitometer (Cat. no: GS800; Quantity One software; Bio‐Rad, Hercules, CA, USA) detected the band intensities with GADPH as reference gene.

### 2.6 TUNEL assay

Apoptosis of treated breast cancer cells was examined using TUNEL assay with the TUNEL kit (Cat. no: 11684817910; Roche; Shanghai, China) adopted. Firstly, after specific treatments, cells underwent a 15 minutes fixation at 4°C with 4% (w/v) paraformaldehyde. Secondly, TUNEL kit was applied for TUNEL staining while the nuclei of cells were dyed for 10 minutes by DAPI (Cat. no: 10236276001; Roche; Shanghai, China). Lastly, the measurement of TUNEL‐positive cells was executed by a fluorescence microscope (Cat. no: CX31‐P; Olympus; Tokyo; Japan).

### 2.7 Cell counting kit‐8 (CCK‐8) assay

With regard to assessing cell viability, Cell Counting Kit (Cat. no: R22305; Dojingdo Molecular Technologies; Rockville, Japan) was employed as instructed. First of all, approximately 2000 cells in triplicate were plated into 96‐well plates. CCK‐8 solution (10 μL) was thereafter put into each well and maintained incubation for 2.5 hours with temperature set at 37°C. Finally, SpectraMax M5 microplate reader (Cat. no: 9232933; QIAGEN; MD, Germany) applied was for measuring the OD value at 450 nm.

### 2.8 5‐ethynyl‐2'‐deoxyuridine (EdU) assay

EdU assay implicating the usage of 5‐ethynyl‐2‐deoxyuridine (EdU) labelling/detection kit (Cat. no: C10310; Ribobio; Guangzhou, Guangdong, China) examined cell proliferation. Breast cancer cells at 5000 cells/well were allowed to grow in 96‐well plates. Post‐transfection of 48 hours, the 96‐well plates were supplied with 50 μmol/L EdU labelling media and further cultured at 37°C containing 5% CO_2_ lasting 2 hours. Prior to cells under the treatment of anti‐EdU working solution, cell was treated by 0.5% Triton X‐100 and 4% paraformaldehyde. The nucleus was labelled by DAPI, and the proportion of EdU‐positive cells was measured after fluorescent microscopy analysing from 5 random fields of view.

### 2.9 Transwell invasion assay

24‐well chambers with 8 μm pore size (Corning, NY, USA) were used for transwell invasion assay in terms of cell invasion detection. BT‐549 or MDA‐MB‐231 cells (with 5 × 10^4^ cells in each well filled with serum‐free media) were placed into the top chamber which is coated with matrigel (Cat. no: 356 234; BD, Franklin Lakes, NJ, USA) at 150‐200 Μl/cm^2^ for 30 minutes at 37°C in advance; the lower chamber was filled with medium which is 10% FBS‐containing. After a 24 h incubation and the removal of cells on the upper chambers, methanol was adopted for the fixing lower membrane surface and Giemsa stained it. At last, a microscope (ZEISS; Oberkochen, Baden, Wurttemberg, Germany) at 200× magnification was put into use for cell counting.

### 2.10 Wound healing assay

MDA‐MB‐231 and BT‐549 cells post‐transfection were placed in 6‐well plates with each well containing 5 × 10^5^ cells. Production of the wounds was implemented with a sterile 200 μL plastic pipette tips scraping cell layer. These cells were further cultivated with medium supplied with 1% FBS, permitting cells migrating into the denuded area. 36 hours later, microscope (Leica, Beijing, China) at 50× magnification was used for acquiring images.

### 2.11 Nuclear/cytoplasmic fractionation

In order to separate cytoplasmic and nuclear RNA in BT‐549 and MDA‐MB‐231 cells, the two fractions including cytoplasm and nuclear of these cells were segmented as directed by the protocols of a PARIS kit (Cat. no: AM1921; Life Technologies; Carlsbad, CA, USA). Following qRT‐PCR (SYBR Premix Ex Taq; TaKaRa; Dalian, Liaoning, China) estimated the extracted RNAs from the cytoplasmic and nuclear fractions.

### 2.12 Fluorescent in situ hybridization (FISH)

Ribobio (Guang Zhou, China) supplied relevant FISH probes and FISH assay was performed following the procedures of a FISH kit (Cat. no: C10910; Ribobio; Guang Zhou, Guangdong, China). The 15 minutes fixation of MDA‐MB‐231 and BT‐549 cells was conducted in 4% formaldehyde, followed by being washed by PBS. The fixed cells later underwent Pepsin (1% in 10 mmol/L HCl) treatment and continuously ethanol dehydrating. Subsequently, the dehydrated cells were placed in a hybridization buffer, mixed with the FISH probes and finally incubated. After 2× Saline Sodium Citrate (SSC) washing, 6‐diamidino‐2‐phenylindole (DAPI) was used for cell staining in the dark. A fluorescence microscope (Zeiss, Oberkochen, Baden, Wurttemberg, Germany) captured the images of cells.

### 2.13 RNA immunoprecipitation (RIP) assay

The AGO2‐RIP experiments with the help of EZ‐Magna RIP Kit (Cat. no: 17‐701; Millipore; Billerica, MA, USA) were executed in BT‐549 and MDA‐MB‐231 cells. Briefly, cells were lysed using RIP lysis buffer with proteinase and RNase inhibitors (Cat. no: 5.05855.0001; Millipore; Billerica, MA, USA), and the RIP lysates were incubated with RIP buffer containing magnetic beads conjugated with human anti‐Ago2 antibody or nonspecific mouse IgG antibody (Cat. no: PP40; Millipore; Billerica, MA, USA). Each immunoprecipitate was digested with proteinase K, and the immunoprecipitated RNAs were subjected to RT‐PCR and gel‐staining analyses to detect circ‐TFF1 enrichment. Each RIP assay was repeated three times.

### 2.14 RNA pull‐down assay

In short, the cell lysates extracted from BT‐549 and MDA‐MB‐231 cells have undergone a 2 hours incubation at 25°C with biotin (Bio)‐labelled oligonucleotide probes specifically against circ‐TFF1 (RiboBio; Guangzhou, Guangdong, China) and Streptavidin‐coupled Dynabeads (Cat. no: 11206D; Invitrogen; Carlsbad, CA, USA) was employed for capturing the circ‐TFF1‐associated miRNA complexes. After which beads/RNA complexes passed through with 1 hour of RIP wash buffer (20‐156; Millipore; Billerica, MA, USA) incubation, which was added with proteinase K at 25°C. qRT‐PCR analysis of the pull‐down RNAs was conducted.

### 2.15 Luciferase reporter assay

The wild‐type or mutated circ‐TFF1 possessing miR‐326 binding sites were obtained from Invitrogen; Carlsbad, CA, USA, which was later subcloned into the pmirGLO Vector (Cat. no: E1330; Promega; Madison, WI, USA) with the aid of T4 DNA Ligase Master Mix (Cat. no: IVGN2108; Thermo Fisher Scientific; Inc, Waltham, MA, USA). Then, miR‐326 mimic or its control, circ‐TFF1‐WT vector or circ‐TFF1‐MUT vector were co‐transfected into indicated breast cancer cells adopting a Nepa21 pulse generator. After transfection for 24 hours, the luciferase activity following a 24 hours transfection was monitored by the Dual‐Glo® Luciferase Assay System (Cat. no: E2920; Promega; Madison, WI, USA) as per the manufacturer's directions.

### 2.16 Tumour formation and in vivo metastasis assay

Three BALB/C nude mice aged 4‐6 weeks (male) was commercially bought from Animal Center of Nanjing University (Nanjing, Jiang su, China) and well bred in a pathogen‐free cabinet. One million of BT‐549 cells, respectively, transfected with two groups either sh‐NC or sh‐circ‐TFF1 plasmids were injected into separate sides of the mouse posterior. The xenograft tumour proliferation was detected every 2 days, and calculation of the tumour volume is following the formula: length × width^2^ × 0.5. 4 weeks later, the mice were sacrificed and tumours were cut off for further analysis. The study strictly followed and conducted under requirements of the National Institutes of Health (NIH) as for animal experiments and the obtained approval from the Animal Experimental Ethics Committee of Nanjing Medical University supported our study.

BT‐549 and MDA‐MB‐231 cells stably transfected with shRNA or sh‐circ‐TFF1 were transplanted into the nude mice via tail vein injection. Five mice were in each group. 8 weeks later, the mice were killed for the collection of lung. The metastatic nodules were calculated after staining and IHC analysis.

### 2.17 Statistical analysis

We conducted experimental statistics analysis with the SPSS and GraphPad Prism 5 software involved. Student's *t* test or chi‐square test was implicated in estimating the disparities between two related groups, whereas differences among three groups or more were evaluated by one‐way analysis of variance (ANOVA). The Pearson correlation coefficients assessed the relations among the expression of circ‐61825, miR‐326 and TFF1. *P* < .05 was considered as the level of statistical significance. All experimental data are presented as means ± standard error of the mean (SEM).

## 3 RESULTS

### 3.1 Circ‐TFF1 and TFF1 were highly expressed in breast cancer

**Figure 1:**
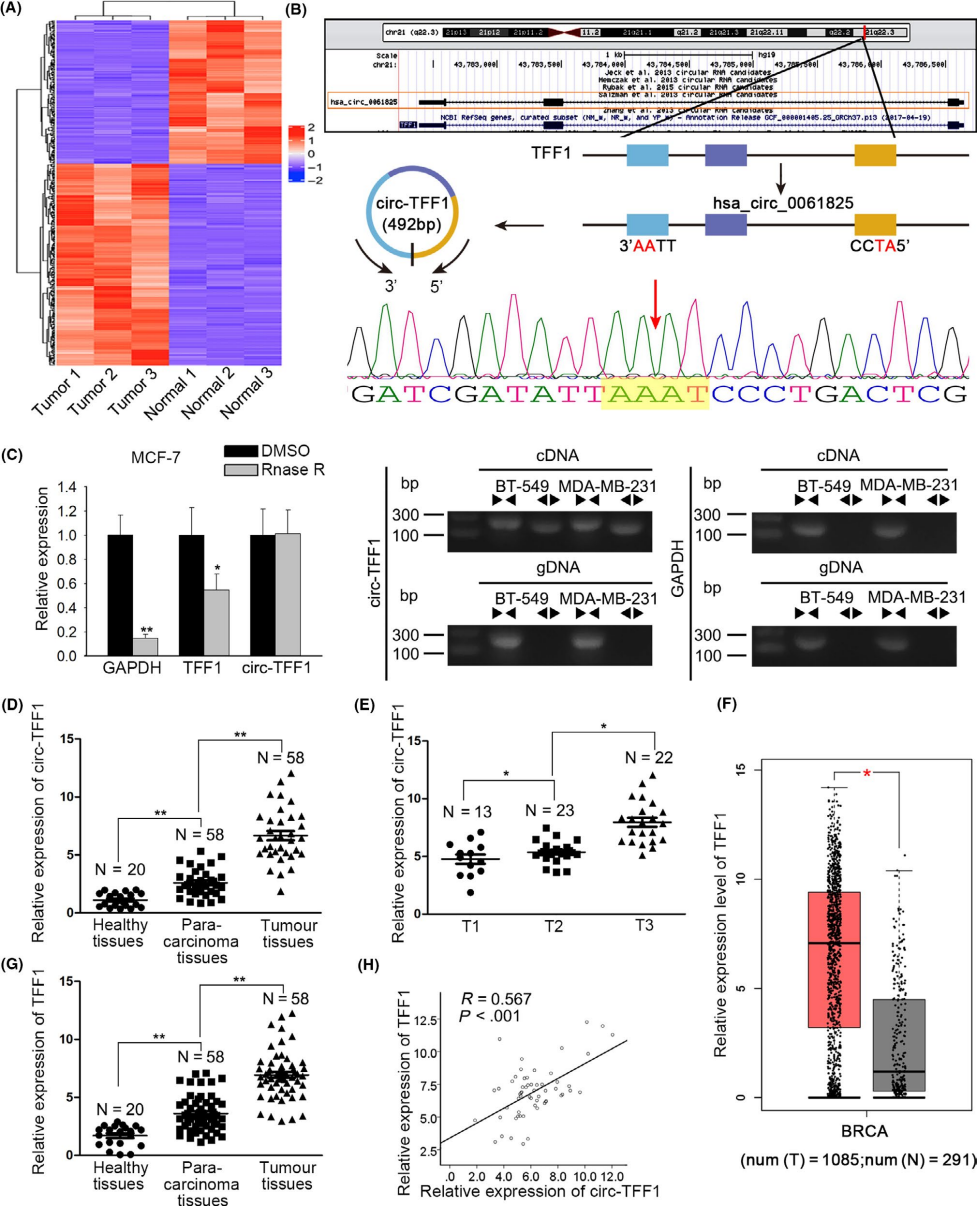
Hsa_circ_0061825 (circ‐TFF1) and TFF1 were both upregulated in breast cancer and in positive association with each other. A, High‐throughput sequencing of circRNAs in tumour and normal tissues. B, The position of circ‐TFF1 in chromosome. C, The circular structure of circ‐TFF1 was verified by RNase R treatment, divergent primer PCR and Sanger sequencing. D‐E, qRT‐PCR results of circ‐TFF1 expression in healthy, para‐carcinoma and tumour tissues and in T1, T2 and T3 stages. F, The high expression of TFF1 in breast carcinoma tissues was gained by TCGA database. G, qRT‐PCR was used for detecting TFF1 expression in healthy, tumorous and non‐tumour tissues. H, Pearson's correlation analysis was utilized for the association between circ‐TFF1 and TFF1. *P < .05, **P < .01

In order to investigate the expression profile of circRNAs in breast cancer, microarray analysis was performed on three pairs of breast cancer tissues and adjacent non‐tumour samples. Among 10 differentially expressed circRNAs with the most significant fold changes, we selected hsa_circ_0061825 (circ‐TFF1) for in‐depth study (Figure 1A). As displayed in Figure 1B, it was indicated by UCSC database that circ‐TFF1 was derived from the exons of the host gene TFF1, which implied the potential administration of circ‐TFF1 on TFF1. In addition, the circular structure of circ‐TFF1 was validated as it presented more stable resistant to RNase R and could only be amplified in cDNA by divergent primers, which was further testified by sequencing (Figure 1C). Subsequently, we detected circ‐TFF1 expression in 58 pairs of clinical tissues and results demonstrated the expression pattern of circ‐TFF1 in healthy tissues, para‐carcinoma tissues and breast cancer samples are low, middle, high (Figure 1D). Importantly, we discovered that the level of circ‐TFF1 was heightened along with the progression of breast cancer (Figure 1E). By analysis of TCGA data, it was found that TFF1 was intensively expressed in tumours from breast cancer patients compared to the normal tissues (Figure 1F), while this result was also confirmed in the collected clinical samples in this study (Figure 1G). Significantly, we revealed that TFF1 expression was positively associated with circ‐TFF1 level in breast cancer tissues (Figure 1H). Taken together, the upregulated circ‐TFF1 and its host gene TFF1 were positively correlated in their expression in breast cancer tissues.

### 3.2 Circ‐TFF1 positively regulated the expression of TFF1 in breast cancer cells

**Figure 2:**
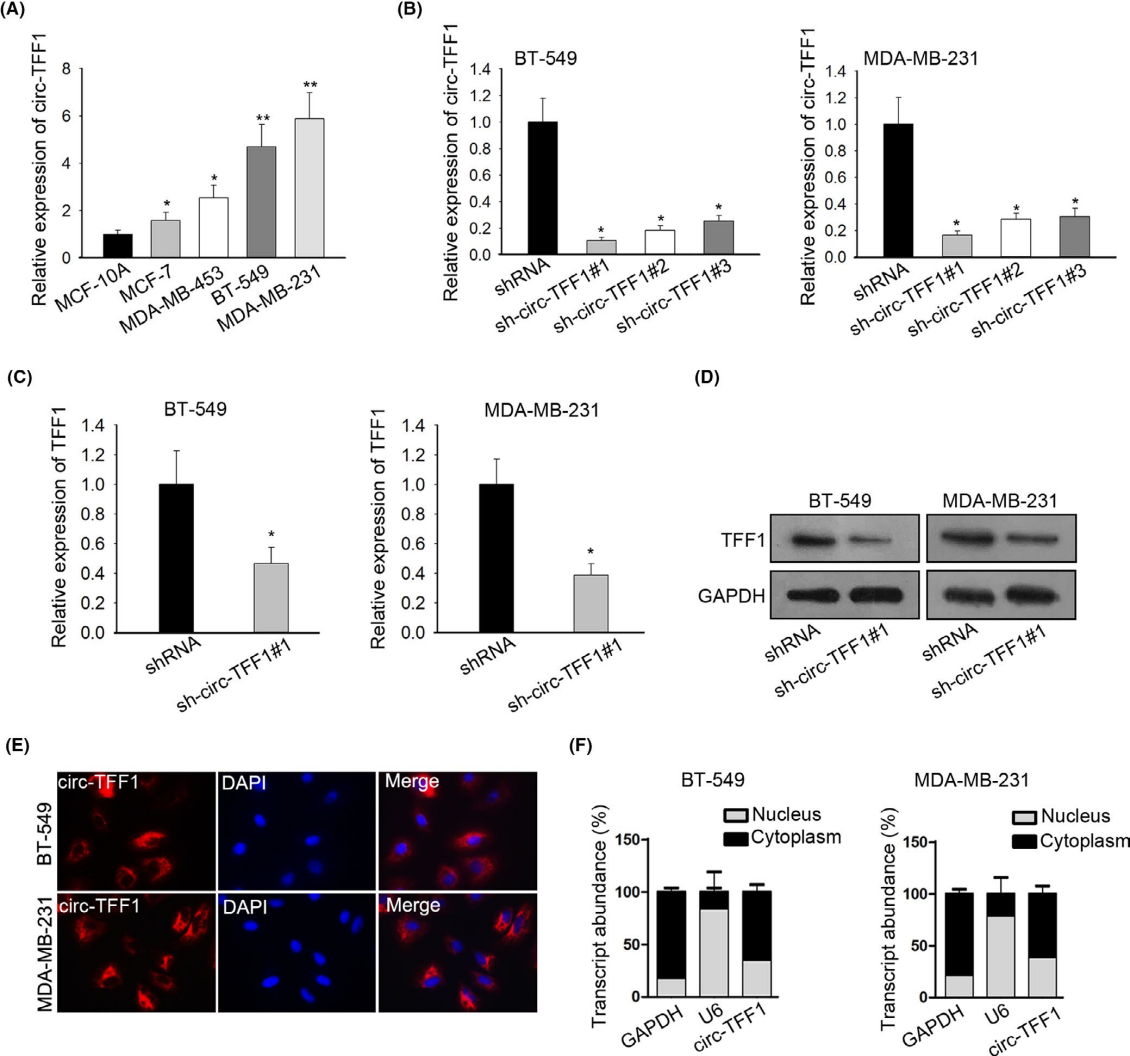
circ‐TFF1 modulated TFF1 positively. A, In breast cancer cells including MDA‐MB‐231, BT‐549, MCF‐7 and MDA‐MB‐453 and normal breast epithelial cells MCF‐10A, circ‐TFF1 expression was examined by qRT‐PCR. B, The transfection efficiency of sh‐circ‐TFF1#1/2/3 in BT‐549 and MDA‐MB‐231 cells was confirmed through qRT‐PCR. (C‐D) TFF1 expression affected by sh‐circ‐TFF1#1 was evaluated by qRT‐PCR and Western blotting. E‐F, Assays of FISH and qRT‐PCR after subcellular fractionation were adopted for the location of circ‐TFF1 in breast cancer cells. *P < .05, **P < .01

Then, we measured the expression of circ‐TFF1 in breast cancer cells and verified that circ‐TFF1 level in breast cancer cells was higher than in normal cells (Figure 2A). To further explore the relationship between circ‐TFF1 and TFF1 in breast cancer cells, the expression of circ‐TFF1 was then silenced in BT‐549 and MDA‐MB‐231 cells which showed highest endogenous circ‐TFF1 expression. As indicated in Figure 2B, all the transfection of three kinds of shRNAs targeting circ‐TFF1 resulted in remarkable reduction on circ‐TFF1 expression in above two cells. Besides, cells transfected with sh‐circ‐TFF1#1 were selected for subsequent assays due to the highest knockdown efficiency. As anticipated, suppression of circ‐TFF1 contributed to the decreased expression of TFF1 at both mRNA and protein levels (Figure 2C,D). Furthermore, the results of FISH assay illuminated that circ‐TFF1 was principally expressed in the cytoplasm of two breast cancer cells (Figure 2E), which was also affirmed by analysing its expression in the cytoplasm and nucleus of breast cancer cells after subcellular fractionation (Figure 2F). To sum up, these results suggested that TFF1 was positively modulated by circ‐TFF1 in breast cancer cells.

### 3.3 Silencing of circ‐TFF1 hindered cell proliferation and promoted apoptosis of breast cancer cells

**Figure 3:**
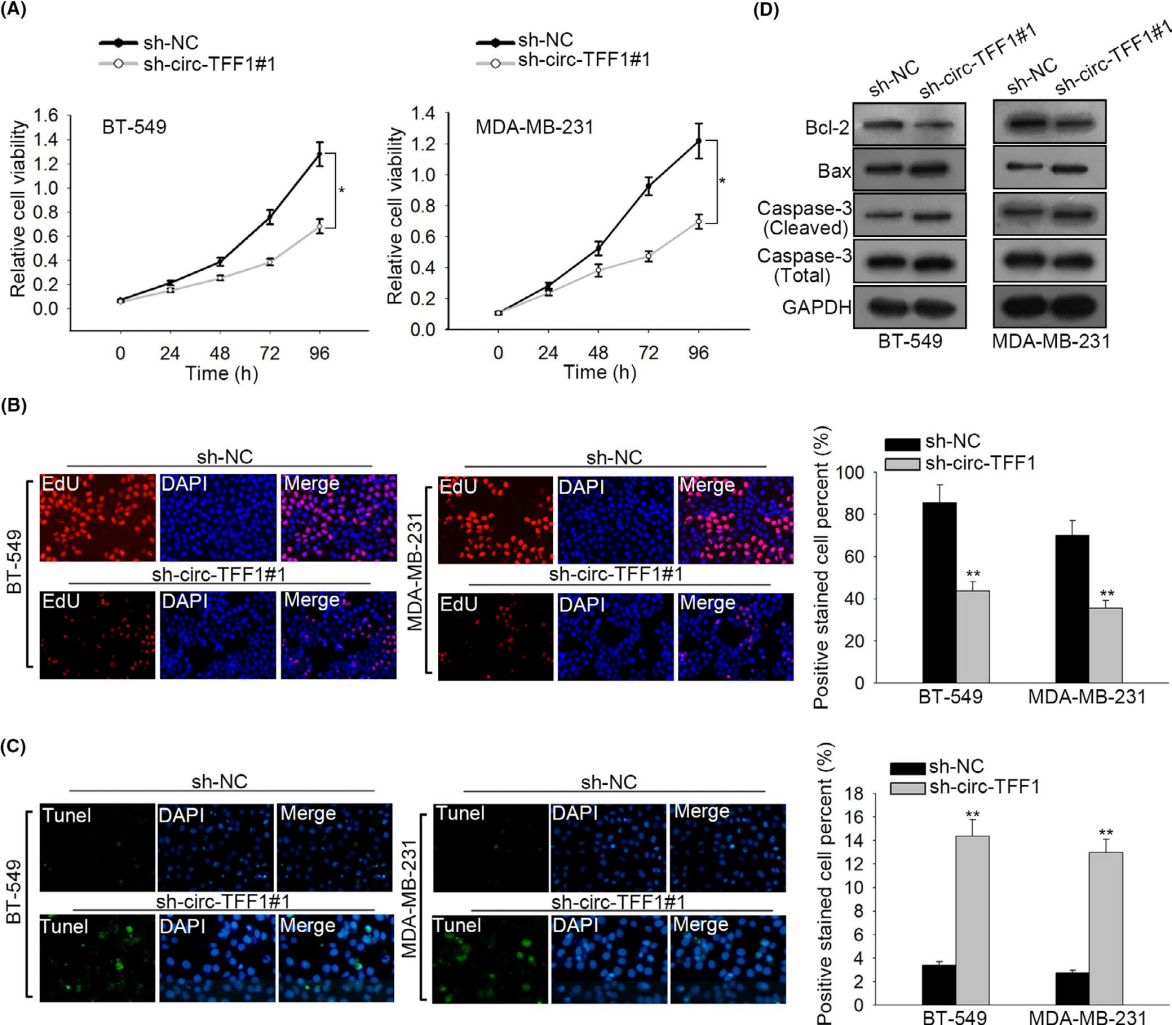
Silencing of circ‐TFF1 inhibited the proliferation and induced the apoptosis in breast cancer. A‐B, CCK‐8 and EdU analyses of cell proliferation in BT‐549 and MDA‐MB‐231 cells transfected with sh‐NC or sh‐circ‐TFF1#1. C, Tunel analysis of cell apoptosis affected by sh‐circ‐TFF1#1 in BT‐549 and MDA‐MB‐231 cells. D, Levels of cell apoptosis‐related proteins (Bcl2, Bax, caspase3 (cleaved), total‐caspase3) were assessed through Western blot. *P < .05, **P < .01

Next, loss‐of‐function assays were implemented to disclose the role of circ‐TFF1 in breast cancer. CCK8 assay delineated that knockdown of circ‐TFF1 repressed the viability of BT‐549 and MDA‐MB‐231 cells (Figure 3A). Consistently, EdU assay showed that the proportion of EdU‐positive cells was dropped by circ‐TFF1 depletion (Figure 3B). Tunnel staining assay unveiled that the overt increase of cell apoptosis was observed in BT‐549 and MDA‐MB‐231 cells stably transfected with sh‐circ‐TFF1#1 (Figure 3C). Besides, Western blot data manifested that suppression of circ‐TFF1 resulted in the declined Bcl‐2 expression and the enhanced levels of Bax and cleaved caspase‐3 (Figure 3D). On the whole, circ‐TFF1 knockdown inhibited cell proliferation but encouraged apoptosis in breast cancer.

### 3.4 Circ‐TFF1 depletion restrained cell migration, invasion and EMT in breast cancer

**Figure 4:**
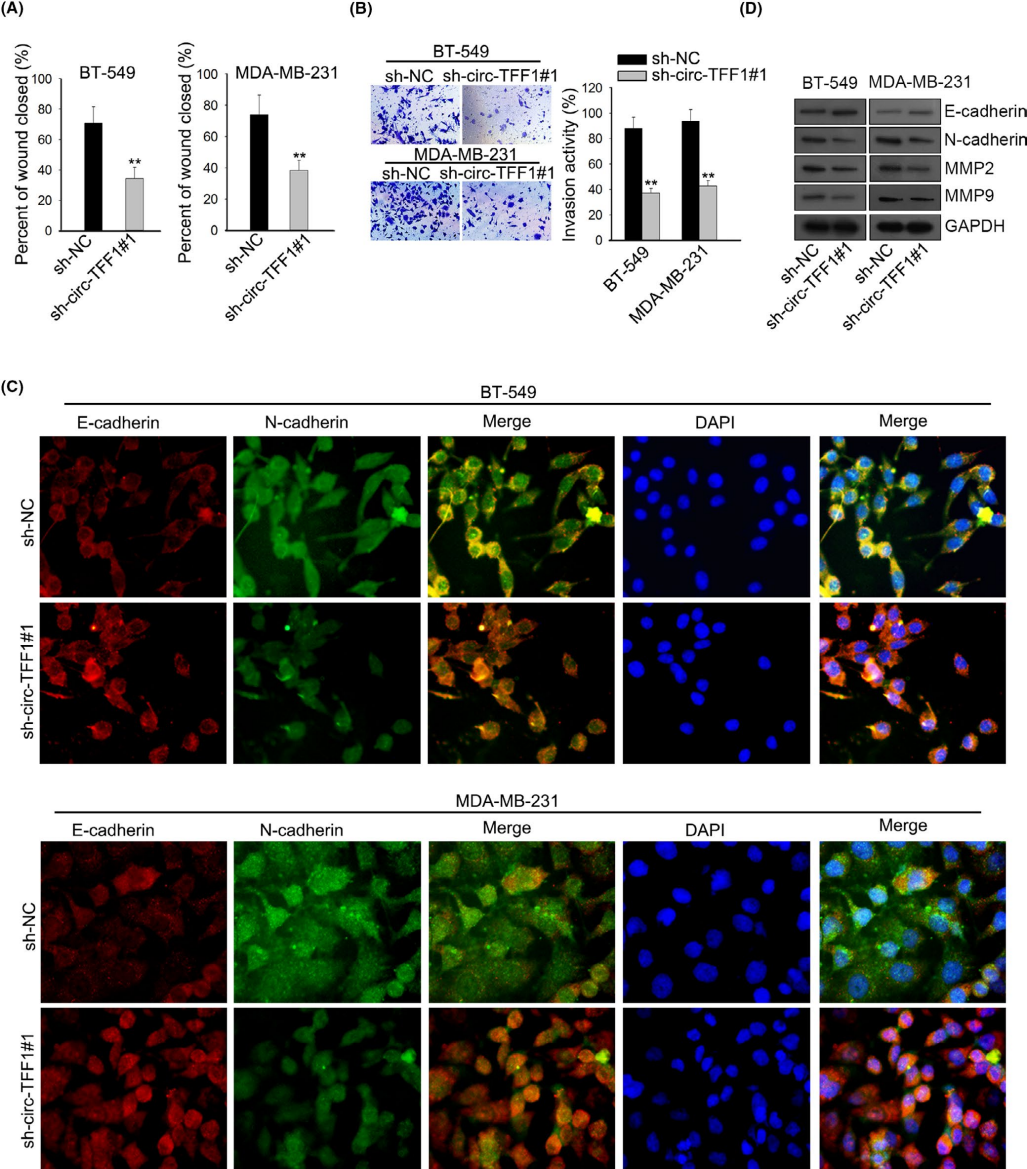
Silencing of circ‐TFF1 repressed metastasis and EMT process of breast cancer. A‐B, The migration and invasion abilities of two cells were separately estimated through would healing assay and transwell assay. C, Levels of N‐cadherin and E‐cadherin by sh‐circ‐TFF1#1 in two cells were detected via IF assay. D, Western blot was performed to test EMT‐related proteins involving N‐cadherin, E‐cadherin, MMP2 and MMP9. **P < .01

To estimate the effects of circ‐TFF1 on cell metastasis, we wondered whether circ‐TFF1 had an impact on cell motility and EMT in vitro. As presented in Figure 4A, the per cent of wound closure was repressed due to silencing of circ‐TFF1. Similarly, transwell assay elucidated that circ‐TFF1 knockdown led to the inhibition of cell invasive capacity (Figure 4B). Immunofluorescence assay expounded the remarkable augment of E‐cadherin level and the significant reduction of N‐cadherin expression in circ‐TFF1‐downregulated cells (Figure 4C). In agreement of findings above, results of Western blot validated the increased expression of E‐cadherin and the declined levels of N‐cadherin, MMP2 and MMP9 occurred with circ‐TFF1 depletion (Figure 4D). By the large, these findings clarified that knockdown of circ‐TFF1 caused the obstruction of cell migration, invasion and EMT in breast cancer.

### 3.5 Circ‐TFF1 upregulated TFF1 expression through sponging miR‐326

**Figure 5:**
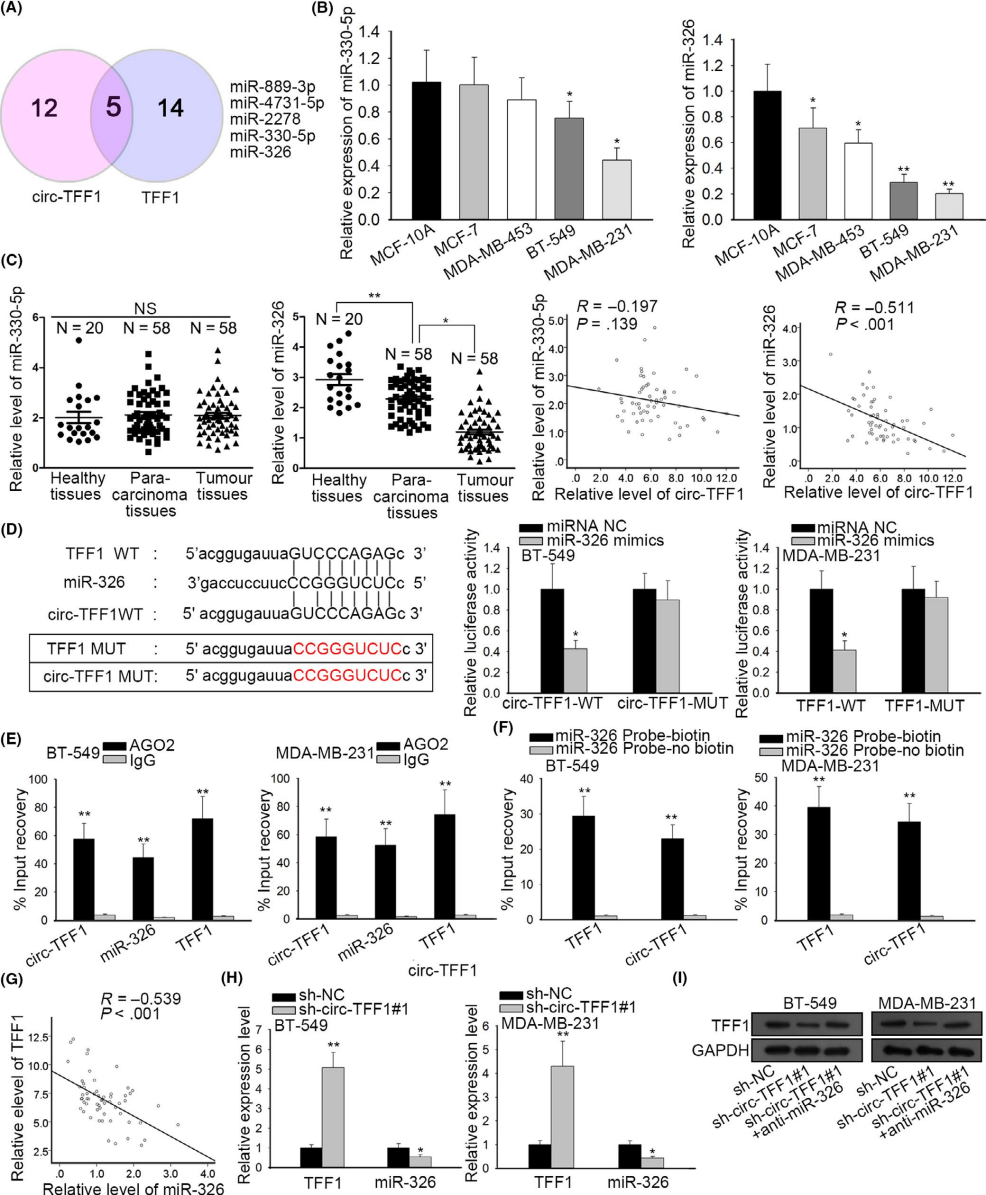
Circ‐TFF1 targets miR‐326 to promote TFF1 expression. A, Five miRNAs shared by circ‐TFF1 and TFF1 were acquired from starBase. The Venn diagram was exhibited. B‐C, The respective levels of miR‐330‐5p and miR‐326 in breast cancer cells and breast epithelial cells, as well as in healthy, para‐carcinoma and tumour tissues were analysed by qRT‐PCR. And the relationship between circ‐TFF1 and miR‐330‐5p or miR‐326 was dissected with Pearson's correlation analysis. D, The wild‐type and mutant binding sites of circ‐TFF1 or TFF1 for miR‐326 were constructed. The interaction between miR‐326 and circ‐TFF1 or TFF1 was confirmed by luciferase reporter assay in 293T cells. E‐F, The relationship among circ‐TFF1, miR‐326 and TFF1 was validated using RIP and RNA pull‐down assays. G, Pearson's correlation analysis was utilized for exploring the association of miR‐326 with TFF1. H, qRT‐PCR detection of the impact of sh‐circ‐TFF1#1 on TFF1 and miR‐326. I, Western blot results of TFF1 expression under transfection of sh‐circ‐TFF1#1 or co‐transfection of sh‐circ‐TFF1#1 and miR‐326 inhibitor. *P < .05, **P < .01

With the assistance of bioinformatics analysis, we discovered that there were 5 miRNAs potentially binding with both circ‐TFF1 and TFF1 (Figure 5A). For the sake of further screening, qPCR assay was applied for examination of miRNA expression in breast cancer cells and suggested that only miR‐326 was downregulated in all of breast cancer cells and miR‐330‐5p was also low‐expressed in only BT‐549 and MDA‐MB‐231 cells, with limited downregulation in the expression of other miRNAs, compared to that in normal cells (Figure 5B and Figure S1A). By measuring the levels of miR‐330‐5p and miR‐326 in clinical tissues, it was verified that only miR‐326 expression in breast cancer tissues was dramatically lower than in healthy and para‐carcinoma samples, and the negative association between miR‐326 and circ‐TFF1 was also demonstrated by Pearson's correlation analysis (Figure 5C). Based on these results, miR‐326 was chosen as a follow‐up subject. Then, the result of luciferase reporter assay exposed that ectopic expression of miR‐326 only mitigated the luciferase activity of TFF1‐WT and circ‐TFF1‐WT, whereas luciferase activity of TFF1‐MUT and circ‐TFF1‐MUT had no response to miR‐326 overexpression (Figure 5D). Also, RIP experiments unravelled that circ‐TFF1, miR‐326 and TFF1 were enriched in compounds precipitated by anti‐Ago2 antibody (Figure 5E). Concordantly, RNA pull‐down assay presented that both circ‐TFF1 and TFF1 were pulled down by miR‐326, proofing the interaction of miR‐326 with TFF1 and circ‐TFF1 (Figure 5F). And we found miR‐326 expression was negatively correlated with TFF1 level (Figure 5G). Additionally, it was uncovered that silencing of circ‐TFF1 inhibited TFF1 expression while promoted the level of miR‐326 in two breast cancer cells (Figure 5H). More importantly, we proved that the protein expression of TFF1 was decreased by circ‐TFF1 depletion and then recovered under co‐suppression of miR‐326 (Figure 5I). Collectively, circ‐TFF1 sponged miR‐326 to regulate TFF1 expression in breast cancer.

### 3.6 Circ‐TFF1/miR‐326/TFF1 accelerated the development of breast cancer

**Figure 6:**
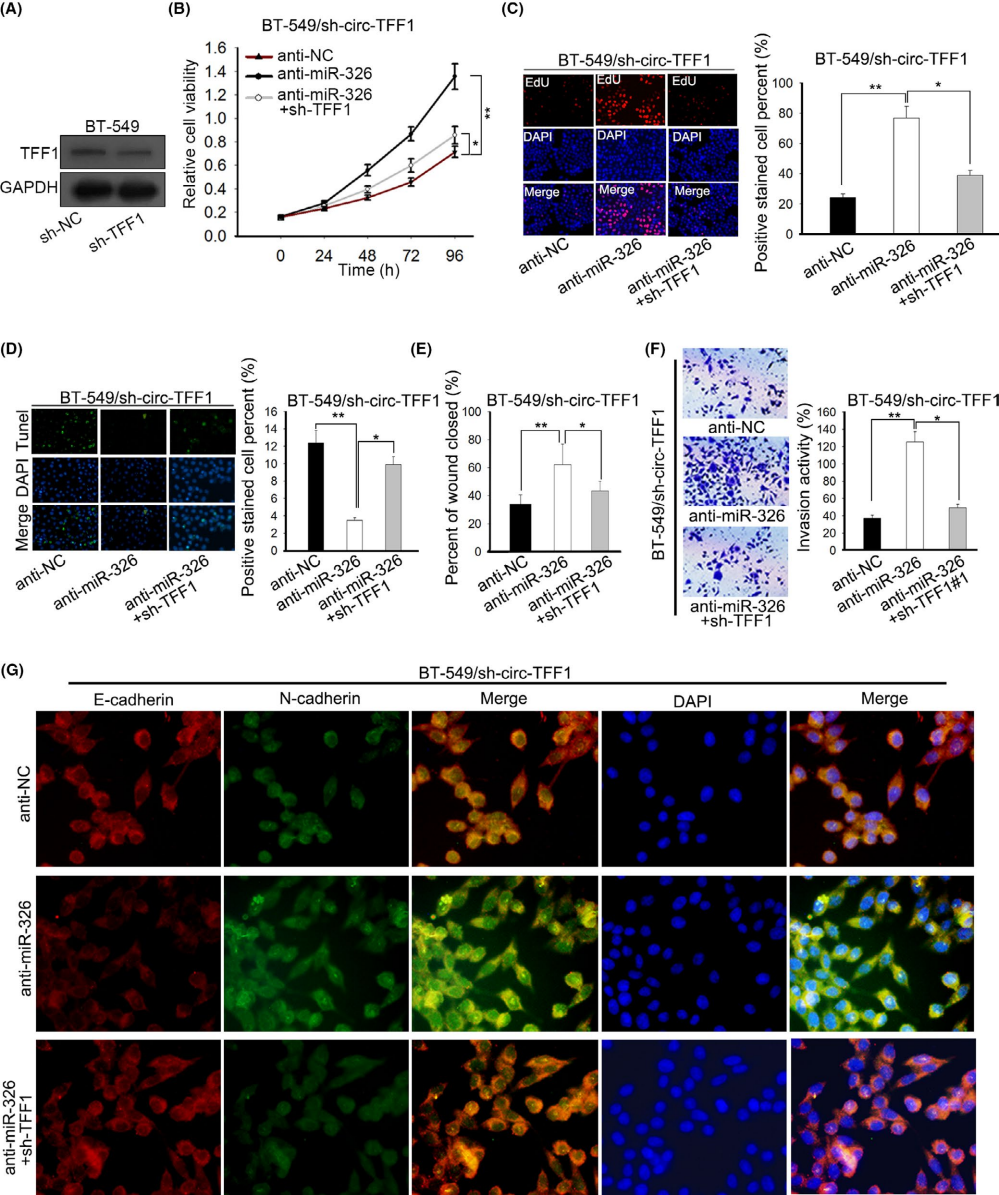
TFF1 downregulation abolished the miR‐326 inhibition‐promoted cellular processes in circ‐TFF1‐silenced BT‐549 cells. A, The inference efficacy of sh‐TFF1#1 was testified by Western blotting. B‐C, Cell proliferation of three indicated groups was individually tested by CCK‐8 and EdU assays. D, Tunel assay was carried out for detecting cell apoptosis in different groups. E‐F, Wound healing and transwell experiments were conducted to estimate cell motility in three groups. G, IF analysis of E‐cadherin and N‐cadherin levels of different groups. *P < .05, **P < .01

In subsequent, rescue experiments were performed to verify the role of circ‐TFF1/miR‐326/TFF1 in breast cancer. Firstly, we confirmed that the expression of TFF1 was knocked down in response to the transfection of shRNAs against TFF1 and BT‐549 cells transfected with sh‐TFF1#1 plasmid exhibited the lowest TFF1 expression (Figure S1B). Also, the declined protein level of TFF1 after transfection was confirmed by Western blot (Figure 6A). As displayed in Figure 6B,C, CCK‐8 and EdU assays unveiled that cell proliferation was boosted by inhibition of miR‐326 and then recovered by TFF1 co‐depletion in circ‐TFF1‐downregulated BT‐549 cells. Conversely, miR‐326 knockdown offset the repression of circ‐TFF1 silence on cell apoptosis in BT‐549 cells, where such counteraction was mitigated facing TFF1 co‐suppression (Figure 6D). Furthermore, circ‐TFF1 depletion‐confined cell migratory and invasive abilities were promoted by miR‐326 inhibition but further restrained owing to knockdown of TFF1 (Figure 6E,F). It was expounded that knockdown of miR‐326 caused the elevated E‐cadherin level as well as the reduced expression of N‐cadherin, and the impacts of miR‐326 depletion on EMT were abolished by TFF1 inhibition in BT‐549 cells stably transfected with sh‐circ‐TFF1 vectors (Figure 6G). Namely, we concluded that circ‐TFF1 executed its function in the progression of breast cancer by modulation of miR‐326/TFF1 axis.

### 3.7 Knockdown of circ‐TFF1 impeded cell growth of breast cancer in vivo

**Figure 7:**
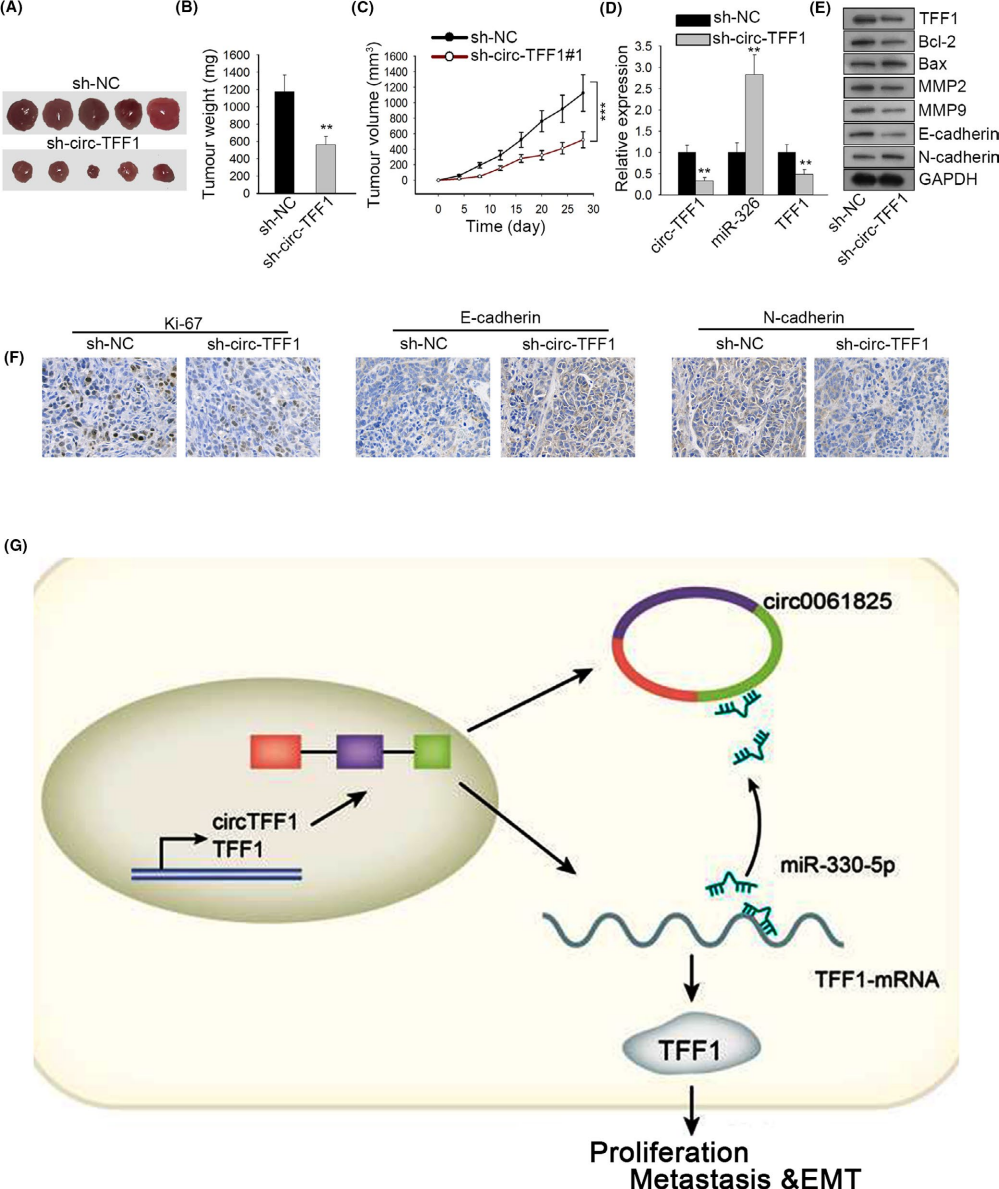
Circ‐TFF1 promotes tumour formation in vivo. A, Images of tumours in sh‐circ‐TFF1#1 or sh‐NC group. B‐C, The difference of tumour weight and the growth curve of tumour volume in two groups were portrayed. D, Expression of circ‐TFF1, miR‐326 and TFF1 in tumours derived from circ‐TFF1‐inhibited BT‐549 cells was determined by qRT‐PCR. E, Western blotting results of the levels of TFF1, Ki67, Bcl‐2, Bax, MMP2, MMP9, E‐cadherin and N‐cadherin when circ‐TFF1 was silenced. F, IHC staining of Ki67, E‐cadherin and N‐cadherin in the paraffin‐embedded sections of tumours collected above. G, The final regulatory circuit of circ‐TFF1‐miR‐326‐TFF1 axis in breast cancer progression. **P < .01, ***P < .001

To further certify the promotion of circ‐TFF1 in breast cancer development, the in vivo animal experiments were conducted through inoculating circ‐TFF1‐silenced BT‐549 cells into mice. We observed that the tumours looked smaller with the weight and size distinctly diminished on account of circ‐TFF1 downregulation (Figure 7A‐C). Meanwhile, we explained that the expression of circ‐TFF1 and TFF1 was reduced while that of miR‐326 increased in tumours from mice with circ‐TFF1‐downregulated BT‐549 cells compared to their levels in those from mice inoculated with control cells (Figure 7D). Moreover, it was indicated by Western blot that silencing of circ‐TFF1 induced a decline in the expression of TFF1, Ki67, Bcl‐2, MMP2, MMP9 and N‐cadherin, but an increase in the levels of Bax and E‐cadherin (Figure 7E), further confirming the inhibitory effects of circ‐TFF1 depletion on cell proliferation, metastasis and EMT in breast cancer. Accordantly, the IHC analysis also suggested that the proliferative marker Ki67 and the mesenchymal marker N‐cadherin were reduced, whereas the epithelial E‐cadherin was enhanced in tumours with downregulated circ‐TFF1 (Figure 7F). BT‐549 and MDA‐MB‐231 cells which were stably transfected with shRNA or sh‐circ‐TFF1 were injected into the body of nude mice. Then, we observed that circ‐TFF1 silence suppressed metastasis in vivo (Figure S2A). In a word, these results validated that suppression of circ‐TFF1 hindered breast cancer cell growth in vivo.

## 4 DISCUSSION

Circular RNAs (circRNAs) are a new class of RNAs with regulatory potency, involving in biological processes of tumours.^22^, ^23^ And circRNAs are involved in the development of carcinomas with various mechanisms. For examples, circRNA_102171 boosts papillary thyroid cancer progression via modulation of CTNNBIP1‐dependent activated β‐catenin pathway,^24^ the circRNA‐ACAP2‐miR‐21‐5p‐Tiam1 feedback loop impacts the proliferation, migration and invasion capacities of SW480 cells,^25^ and circRNA ZNF609 serves as a competitive endogenous RNA of FOXP4 by absorbing miR‐138‐5p in renal carcinoma.^26^ However, the investigation of circRNAs in cancers was still inadequate.

In the current study, we identified ten significantly variable circRNAs in breast cancer and paired normal tissues, and selected hsa_circ_0061825 (circ‐TFF1) due to the highest expression in breast cancer tissues. Meanwhile, we discovered the host gene of circ‐TFF1, TFF1, was also highly expressed in breast cancer tissues, in accordance with previous researches that TFF1 was specifically found to be elevated and promoted the development of breast cancer.^14^, ^15^, ^27^ Besides, we uncovered that TFF1 expression was positively associated with circ‐TFF1 expression in breast cancer tissues. In addition, circ‐TFF1 was prevailingly seated in the cytoplasm of breast cancer cells. Based on these findings, we assumed that circ‐TFF1 might involve in ceRNA network with TFF1. Next, the function role of circ‐TFF1 in breast cancer was inquired. The research was the first to report the tumour‐promoting role of circ‐TFF1 in breast cancer, affecting cell proliferation, apoptosis, migration and invasion, and EMT process.

To inspect whether circ‐TFF1 took part in the ceRNA model, we searched on starBase for the common miRNAs of circ‐TFF1 and TFF1 and eventually identified miR‐326. Mounting evidence has illustrated the tumour‐repressing role of miR‐326. For instances, circPUM1 facilitates the malignance of lung adenocarcinoma via regulating miR‐326^28^; HOTAIR‐miR‐326‐FUT6 axis promotes colorectal cancer progression by modulating fucosylation of CD44 through PI3K/AKT/mTOR pathway^29^; hsa_circ_0003998 accelerates cell proliferation and invasion via absorbing miR‐326 in non–small‐cell lung cancer^30^; and lncRNA H19, in contrast to miR‐326, is overexpressed and predicts poor survival in glioblastoma.^31^ Mechanism assays certified that circ‐TFF1 promotes TFF1 expression by absorbing miR‐326. Furthermore, rescue and in vivo experiments revealed that circ‐TFF1 targets miR‐326 to enhance TFF1 expression for the cellular activities of breast cancer. The participation of circ‐TFF1/miR‐326/TFF1 pathway was firstly displayed in our study.

In summary, this investigation proved that circ‐TFF1, which derived from the host gene TFF1, elicited an oncogenic function in breast cancer by freeing TFF1 from miR‐326‐induced silence (Figure 7G). The link between the modulatory mechanism of circ‐TFF1‐miR‐326‐TFF1 axis and cellular activities in breast cancer is revealed here for the first time, and our findings will offer a novel insight into the therapy of breast cancer patients.

## CONFLICTS OF INTEREST

The authors indicate no conflicts of interest in this study.

## **AUTHOR** **CONTRIBUTIONS**

Gaofeng Pan conceptualized the study and involved in formal analysis and project administration; Anwei Mao contributed to methodology, validated the study and curated the data; Jiazhe Liu investigated the study and contributed software; Jingfeng Lu provided resources; Junbin Ding wrote original draft of the manuscript, visualized the data and acquired funding; Weiyan Liu wrote, reviewed, edited and supervised the study.

## Data Availability

Research data are not shared.
