# Supplementary figures and images for: Circular RNA hsa_circ_0061825 (circ‐TFF1) contributes to breast cancer progression through targeting miR‐326/TFF1 signalling
Source: Cell Prolif. 2020 Jan 21;53(2):e12720. doi: 10.1111/cpr.12720 (PMC7048212; doi:10.1111/cpr.12720)

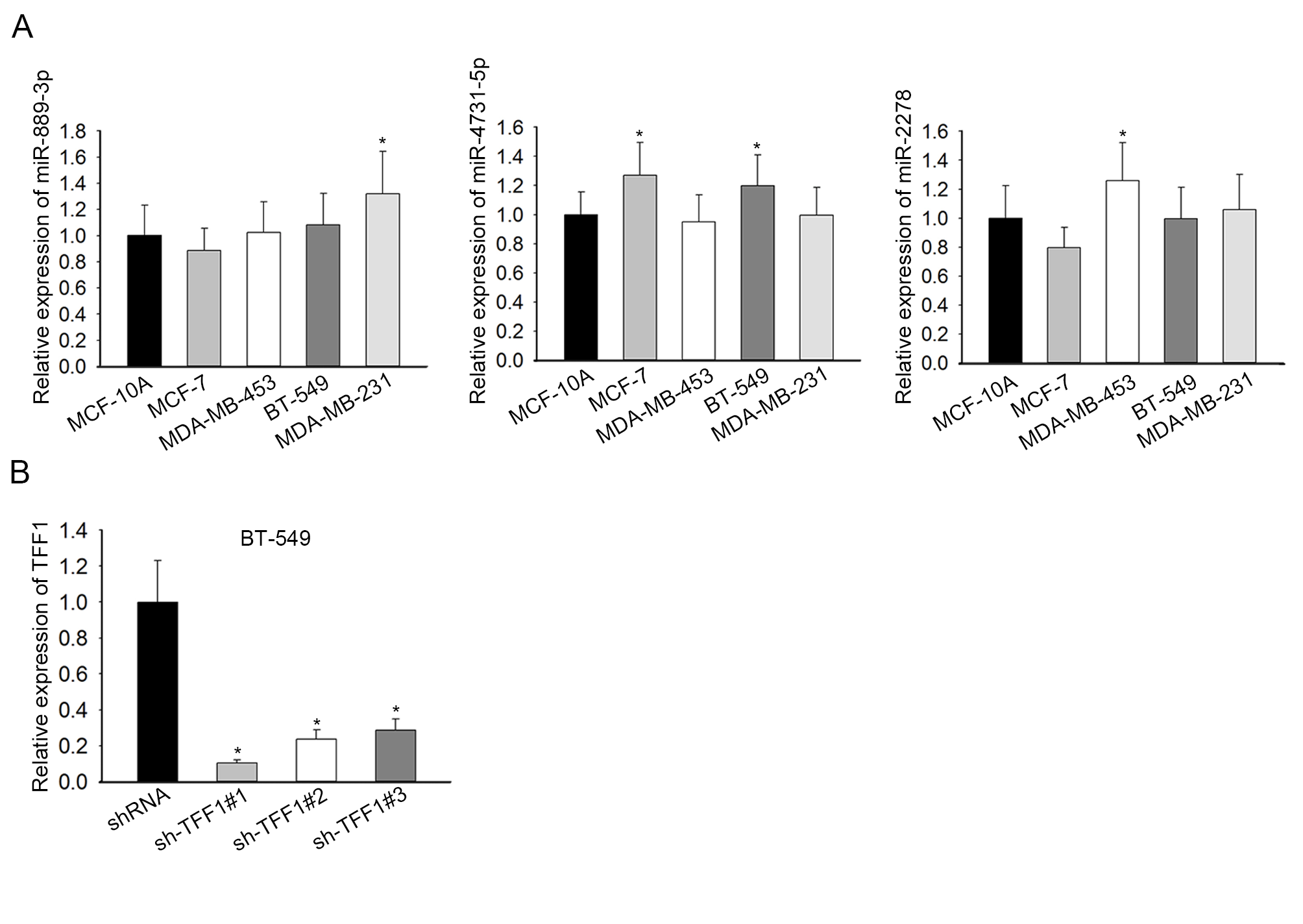

Supplement: Supplementary file 1 [file CPR-53-e12720-s001.tif]

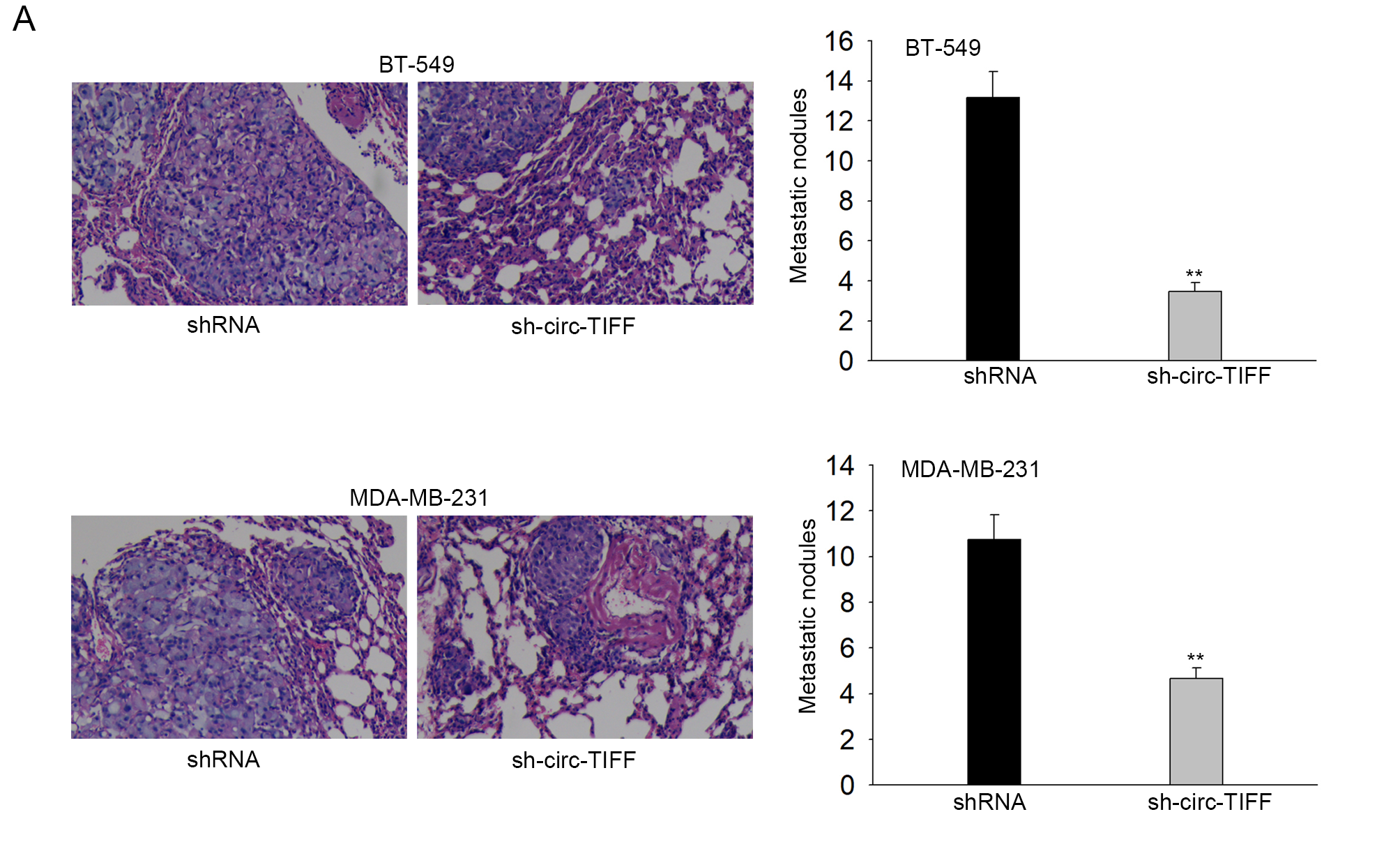

Supplement: Supplementary file 2 [file CPR-53-e12720-s002.tif]
